# Is There a Chronic Elevation in Organ-Tissue Sleeping Metabolic Rate in Very Fit Runners?

**DOI:** 10.3390/nu8040196

**Published:** 2016-04-02

**Authors:** Taishi Midorikawa, Shigeho Tanaka, Takafumi Ando, Chiaki Tanaka, Konishi Masayuki, Megumi Ohta, Suguru Torii, Shizuo Sakamoto

**Affiliations:** 1 College of Health and Welfare, J.F. Oberlin University, 3758 Tokiwamachi, Machida, Tokyo 194-0294, Japan; c-tanaka@obirin.ac.jp; 2 Department of Nutritional Science, National Institute of Health and Nutrition, National Institutes of Biomedical Innovation, Health and Nutrition, 1-23-1 Toyama, Shinjuku-ku, Tokyo 162-8636, Japan; tanakas@nih.go.jp (S.T.); takafumi_andy@yahoo.co.jp (T.A.); 3 Faculty of Sport Sciences, Waseda University, 2-579-15 Mikajima, Tokorozawa, Saitama 359-1192, Japan; m.konishi@aoni.waseda.jp (K.M.); shunto@waseda.jp (S.T.); s.sakamoto@waseda.jp (S.S.); 4 School of International Liberal Studies, Chukyo University, 101 Tokodachi, Kaizu-cho, Toyota, Aichi 470-0393, Japan; m-ohta@lets.chukyo-u.ac.jp

**Keywords:** sleeping energy expenditure, organ-tissue mass, V̇O_2_peak

## Abstract

It is unclear whether the resting metabolic rate of individual organ-tissue in adults with high aerobic fitness is higher than that in untrained adults; in fact, this topic has been debated for years using a two-component model. To address this issue, in the present study, we examined the relationship between the measured sleeping energy expenditure (EE) by using an indirect human calorimeter (IHC) and the calculated resting EE (REE) from organ-tissue mass using magnetic resonance imaging, along with the assumed metabolic rate constants in healthy adults. Seventeen healthy male long-distance runners were recruited and grouped according to the median $\overset{\cdot }{V}$O_2_peak: very fit group (>60 mL/min/kg; *n* = 8) and fit group (<60 mL/min/kg; *n* = 9). Participants performed a graded exercise test for determining $\overset{\cdot }{V}$O_2_peak; X-ray absorptiometry and magnetic resonance imaging were used to determine organ-tissue mass, and IHC was used to determine sleeping EE. The calculated REE was estimated as the sum of individual organ-tissue masses multiplied by their metabolic rate constants. No significant difference was observed in the measured sleeping EE, calculated REE, and their difference, as well as in the slopes and intercepts of the two regression lines between the groups. Moreover, no significant correlation between $\overset{\cdot }{V}$O_2_peak and the difference in measured sleeping EE and calculated REE was observed for all subjects. Thus, aerobic endurance training does not result in a chronic elevation in the organ-tissue metabolic rate in cases with $\overset{\cdot }{V}$O_2_peak of approximately 60 mL/min/kg.

## 1. Introduction

Sleeping energy expenditure (EE) is the minimum EE required to sustain body function. The sleeping EE of the entire body is an important component of total daily energy expenditure and is generally slightly lower than the resting EE (REE) in the lying or sitting position [1]. In cases with low activity-related energy expenditure, sleeping EE accounts for approximately 60%–70% of the total energy expenditure [2,3]. Hence, the variability in sleeping EE is closely associated with energy equilibrium. Moreover, the inter-individual variability in sleeping EE is reported to be small, probably due to the accuracy of using an indirect human calorimeter (IHC) for measurement in a stable condition [1].

Previous studies, which predicted REE by multiplying the mass measured using the magnetic resonance imaging (MRI) method and the assumed constant metabolic rate of each organ-tissue among healthy adults, indicated that the calculated REE from organ-tissue mass was well correlated with the measured REE using indirect calorimetry in young and middle-aged men and women, as well as in underweight and obese healthy adults [4,5,6]. The results suggested that the variability in organ-tissue mass is responsible for the variation in REE, and that the metabolic coefficients per unit organ-tissue mass remain almost constant for untrained adults. Thus, the REE of the entire body can be expressed as summation of the resting energy metabolism from individual organs and tissues in healthy young and middle-aged men and women.

A high sleeping EE and/or REE due to exercise training is likely to contribute to effective weight management, although the supporting evidence is controversial. Based on the REE model at the organ-tissue level, increased skeletal muscle (SM) mass and internal organ mass due to exercise training would be closely related to a high sleeping EE and/or REE. In fact, studies have shown that the high REE (>2000 kcal/day) in Sumo wrestlers, with a fat-free mass (FFM) of approximately 80 kg and $\overset{\cdot }{V}$O_2_max of 30 mL/min/kg, can be attributed to the presence of a larger absolute amount of low and high metabolically active tissue, such as the SM, liver, and kidney [7,8]. Hence, it is highly likely that changes in organ-tissue mass after exercise training can lead to an increase in REE (e.g., 13 kcal/kg/day for SM [9]).

Moreover, the increase in the absolute value of sleeping EE and/or REE via exercise training may also be caused by the enhancement of the resting metabolic rate of individual organ-tissue. A previous study indicated that exercise training results in a significant elevation in REE, without an increase in the FFM; however, it was noted that the time from the termination of the last exercise bout to REE measurement was a significant factor for REE elevation, and that long-term excess post-exercise O_2_ consumption may persist for up to 48 h [10]. In previous cross-sectional studies that did not have problems related to the variability of REE (e.g., age, gender, body composition, and time to measure REE), it was found that the resting metabolic rate per unit of FFM—a simple index of metabolic rate—was not related to the $\overset{\cdot }{V}$O_2_max in young healthy women [11]. Moreover, over the last decade, it was found that aerobic endurance training does not affect REE and the REE adjusted for FFM [12,13], and that endurance exercise training prevents a seasonal decline in the absolute value of REE and the REE/FFM ratio [14]. It is unclear whether the resting metabolic rate of individual organ-tissue in adults with high aerobic fitness is higher than that in untrained adults; in fact, debates on this topic using the two-component model remain unresolved.

Furthermore, many previous studies indicated that variations in REE without increasing organ-tissue mass appear to be correlated with many physiological factors, such as thyroid status, circulating triiodothyronine (T_3_), and thyroxine (T_4_), which increase the metabolism of the various cells, as well as epinephrine and norepinephrine, which increase heat production [10,14,15]. Based on these findings, it is possible that the resting metabolic rate per unit organ-tissue mass is elevated by hormones, the release of which is stimulated by aerobic exercise.

In the present study, we aimed to assess whether there is a chronic elevation in organ-tissue sleeping metabolic rate due to the aerobic fitness level. Therefore, we examined the relationship between the measured sleeping EE by using IHC and the calculated REE from organ-tissue mass using MRI, as well as the assumed metabolic rate constants in healthy adults based on a previously published approach [4], including blood analysis.

## 2. Materials and Methods

### 2.1. Subjects

A total of 17 healthy male Japanese long distance runners aged 18–23 years were recruited for the study. The long distance runners had participated in regular training for an average of five years. None of the subjects had a history of smoking or cardiovascular, endocrine, or orthopaedic disorders; had ever tested positive for anabolic steroids; or had been taking any medication during the measurement period. All the subjects received a verbal and written description of the study and provided informed consent to participate prior to testing. The study protocol was approved by the Ethical Committee of Waseda University and the National Institute of Health and Nutrition, and was conducted according to the guidelines laid down in the Declaration of Helsinki.

### 2.2. $\overset{\cdot }{V}$O_2_peak Measurement

Participants performed a graded exercise test to exhaustion on a treadmill (MAT-2700, Fukuda Denshi, Japan) to determine the $\overset{\cdot }{V}$O_2_peak. The graded exercise test was started at 1 km/h and 0% slope, and the speed and slope of the treadmill were increased every minute by approximately 1 metabolic equivalent until the participants were exhausted [16,17]. During the exercise, a 12-lead electrocardiogram was electronically recorded (Stress Test System ML-6500, Fukuda Denshi, Japan), and the heart rates were derived from the RR interval. Pulmonary gas exchange parameters (oxygen uptake ($\overset{\cdot }{V}$O_2_), carbon dioxide output ($\overset{\cdot }{V}$CO_2_), and respiratory exchange ratio (RER)) were determined in a breath-by-breath assessment using a gas analyser (AE-300S, Minato Medical Science, Osaka, Japan). Maximal metabolic measurements and heart rate were recorded only if the participants met at least two of the following three criteria [18]: (1) achieved $\overset{\cdot }{V}$O_2_ plateau (<150 mL/min) despite increasing the exercise intensity; (2) the highest heart rate measured during the last minute of the exercise was >90% of the predicted maximal heart rate (220 − age (in years)); and (3) the highest RER during the final stage of the incremental exercise was >1.10.

### 2.3. Anthropometry and Dual Energy X-ray Absorptiometry (DXA) Measurements

Body mass was measured on a digital balance to the nearest 0.1 kg, with the subjects wearing only minimal clothing, whereas the height was measured on a stadiometer to the nearest 0.1 cm. Body mass index (kg/m^2^) was calculated as body weight (kg) divided by the square of the height (m). Total fat mass was measured using DXA (Delphi A-QDR, Hologic Inc., Bedford, MA, USA; Version 12.4:3 Auto Whole Body Fan Beam). The estimated coefficient of validation for DXA measurements from test-retest analysis was determined to be <1%.

### 2.4. Measurement of Organ-Tissue Mass Using MRI

The volumes of whole-body SM, the internal organs (liver and kidney), and the brain were measured using a General Electric Signa EXCITE VI 1.5 Tesla scanner (Milwaukee, WI, USA). A T1-weighted spin-echo, axial-plane sequence was employed, with a 500-ms repetition time and a 13.1-ms echo time, during breath holding scans and normal breathing scans. Subjects rested quietly in the magnet bore in the supine position with their hands placed on their abdomen. Contiguous transverse images with 1.0-cm slice thicknesses (0-cm interslice gap) were obtained from the top of the head to the malleolus lateralis for each subject. Approximately five sets of acquisitions were performed, extending from the top of the head to the femoral head, during breath holding (approximately 30 s/set). The other sets of acquisitions were obtained from the femoral head to the malleolus lateralis during normal breathing [19]. All images (approximately 170 slices per person) were traced by a highly trained technician, while including the SM, brain, and abdominal organ segments and excluding connective tissue, blood vessels, and fat tissue. MR images were analysed using ZedView software (LEXI Co., Ltd, Tokyo, Japan) for segmentation and calculation of the cross-sectional tissue areas.

The volumes of the SM, liver, kidney, and brain were calculated from the sum of the cross-sectional area (cm^2^) determined by tracing the images, and then multiplying the value by the slice thickness (1 cm). The volumes (cm^3^) were converted to masses (kg) by using the following densities: 1.041 g/cm^3^ for the SM [20], 1.060 g/cm^3^ for liver, 1.050 g/cm^3^ for kidney, and 1.036 g/cm^3^ for brain [21]. The estimated coefficient of validation (CV) for SM volume measurements from a test-retest analysis was determined to be 2% [19]. The percentage of difference in measurements for the same scan on two separate days by the same technician was 0.3% for the liver, 0.5% for the kidney, and 0.6% for the brain (*n* = 5).

Since the constantly pulsing heart produced artifacts, heart mass (g) was estimated from the height and body mass by using the following formula: 22.81 × height (m) × body mass^0.5^ (kg) − 4.15 [22]. Adipose tissue mass was calculated from fat mass with the assumption that 85% of the adipose tissue was fat and 15% of the adipose tissue consisted of the remaining calculated fat-free component [23]. Residual mass was calculated as the total body mass minus the sum of the SM, adipose tissue, brain, liver, kidney, and heart masses, as the total body mass was considered to include the sum of the organ masses. Accordingly, residual mass was considered to be composed of bone, blood, skin, intestine, connective tissue, and lung tissue [20].

### 2.5. Measurement of Sleeping EE Using IHC

The subjects entered the IHC chamber at 18:00–19:00 on the study day, had dinner at 18:30 or 19:00, went to bed at 23:00 after sedentary activities, and slept until 07:00 the following morning [1]. Each subject was provided a standardized dinner to meet their EE during their stay in the chamber based on the predicted basal metabolic rate and an assumed physical activity level of 1.5 [1]. None of the subjects performed any exercise for >48 h prior to testing. Details of the IHC method have been previously published [24,25]. In brief, the respiratory chamber comprised an air-tight room (20,000 or 15,000 L) that was equipped with a bed, desk, chair, television with video deck, compact disc player, telephone, toilet, and sink. The temperature and relative humidity in the room were controlled at 25 °C and 55%, respectively. The O_2_ and CO_2_ concentrations (maintained by air supply and exhaust) were measured using mass spectrometry. For each experiment, the gas analyser (ARCO-1000A-CH; Arco System, Inc., Kashiwa, Japan) was initially calibrated using a certified gas mixture and atmospheric air. The flow rate of exhaust from the chamber was assessed using a pneumotachograph (FLB1; Arco System, Inc., Kashiwa, Japan). The flow meter was calibrated before each measurement, and the flow rate was maintained at ~60 L/min. $\overset{\cdot }{V}$O_2_ and $\overset{\cdot }{V}$CO_2_ were determined based on the flow rate of exhaust from the chamber as well as the concentrations of the inlet and outlet air from the chamber, respectively [26]. The values of $\overset{\cdot }{V}$O_2_ and $\overset{\cdot }{V}$CO_2_ were recorded under conditions of standard temperature and pressure and under dry conditions. EE was estimated from $\overset{\cdot }{V}$O_2_ and $\overset{\cdot }{V}$CO_2_ using Weir’s equation [27]. The accuracy and precision of the IHC for measuring EE, as determined by the alcohol combustion test, was 99.8% ± 0.5% (mean ± standard deviation (SD)) in 6 h and 99.4% ± 3.1% in 30 min. Sleeping EE was defined as the minimum EE recorded over 3 h of sleep between 23:00 and 07:00.

### 2.6. Calculation of REE

REE was calculated as the sum of seven body compartments (SM, adipose tissue, brain, liver, kidney, heart, and residual mass) multiplied by the corresponding tissue-respiration rate, which is based on specific reported tissue-metabolic rates [9]. The calculated REE was computed using the following equation [4]: calculated REE (kcal/day) = (13 × SM mass) + (4.5 × adipose tissue mass) + (240 × brain mass) + (200 × liver mass) + (440 × kidney mass) + (440 × heart mass) + (12 × residual mass).

### 2.7. Blood Collection and Analysis

Blood samples were collected into tubes containing thrombin for analysing the levels of thyroid-stimulating hormone (TSH), total and free T_3_, and total and free T_4_, as well as into tubes containing EDTA-2Na for analysing the levels of plasma epinephrine and norepinephrine. The blood samples in both tubes were centrifuged (3000 rpm for 10 min) after collection, and the serum and plasma were transferred into plastic tubes. Serum and plasma samples were immediately stored at a temperature of <−30 °C until further analysis. The concentrations of serum TSH and total and free T_3_ and T_4_ were measured using an electrochemiluminescence immunoassay. Plasma epinephrine and norepinephrine concentrations were measured using high-performance liquid chromatography. Blood samples were analysed by SRL, Inc. (Tokyo, Japan).

### 2.8. Statistical Analysis

Results are expressed as means ± standard deviation for all variables. A total of 17 subjects were assigned to two groups according to the median $\overset{\cdot }{V}$O_2_peak of 60 mL/min/kg: very fit group (>60 mL/min/kg; *n* = 8) and fit group (<60 mL/min/kg; *n* = 9). The differences between the two groups were tested for significance by using an unpaired *t*-test, and were illustrated by using a box-plot diagram. Pearson’s product-moment analysis was used to compare the measured sleeping EE and calculated REE in the very fit and fit runners, respectively. The difference in the slopes and intercepts of the regression lines were tested between very fit runners and fit runners by using analysis of covariance. Bland-Altman analysis was conducted by plotting the differences in the measured sleeping EE and calculated REE values against the corresponding mean values [28]. In all the subjects, Pearson’s product-moment analysis was used to assess the relationship of $\overset{\cdot }{V}$O_2_peak with the difference in measured sleeping EE and calculated REE. Statistical analyses were performed using SPSS for Windows (IBM SPSS version 22.0; SPSS Inc., Chicago, IL, USA). Differences were regarded as significant when the probabilities were <0.05.

## 3. Results

There was no difference in mean age, standing height, body mass, BMI, fat mass, and FFM between very fit and fit runners, although body fat percentage was different between these groups; moreover, very fit runners had a higher $\overset{\cdot }{V}$O_2_peak as compared to the fit runners (Table 1). Organ-tissue mass (SM, adipose tissue, brain, liver, kidney, heart, and residual mass) and the ratio of SM mass (45.9% *vs.* 45.9%), brain (2.9% *vs.* 3.1%), liver (2.8% *vs.* 2.6%), and kidney (0.6% *vs.* 0.6%) to FFM were also similar between very fit and fit runners (Table 2). Moreover, the measured sleeping EE to FFM ratio (26.7 ± 2.0 kcal/kg/day) in very fit runners was not significantly different from that in fit runners (28.4 ± 2.3 kcal/kg/day; *p* = 0.07).

**Figure 1:**
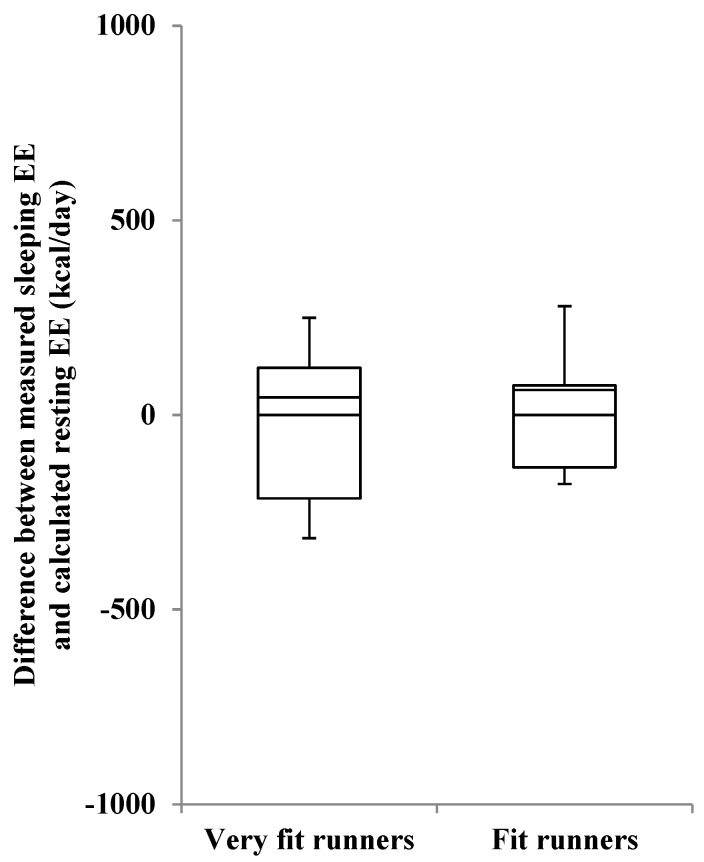
Box-plot diagram for comparing measured sleeping energy expenditure and estimated resting energy expenditure. EE: energy expenditure; REE: resting energy expenditure.

**Figure 2:**
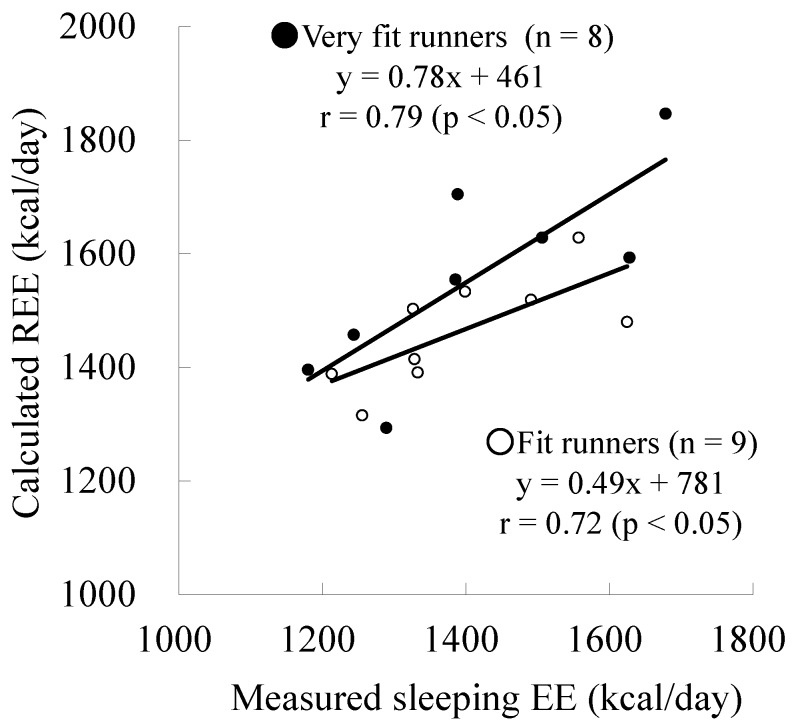
Relationship between the measured sleeping energy expenditure and calculated resting energy expenditure. EE: energy expenditure; REE: resting energy expenditure.

**Figure 3:**
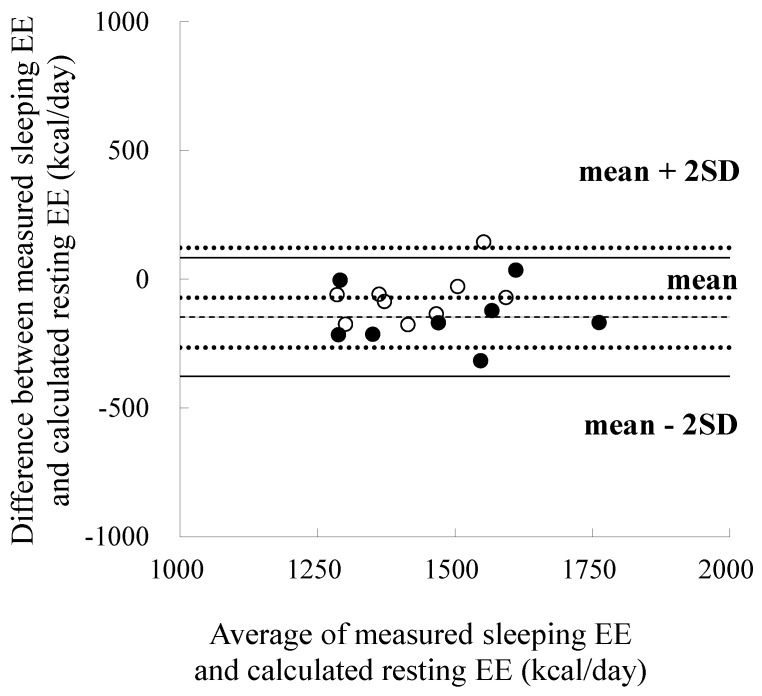
Bland-Altman analysis for comparing measured sleeping energy expenditure and estimated resting energy expenditure. EE: energy expenditure; REE: resting energy expenditure; ●: very fit runners; ○: fit runners.

**Figure 4:**
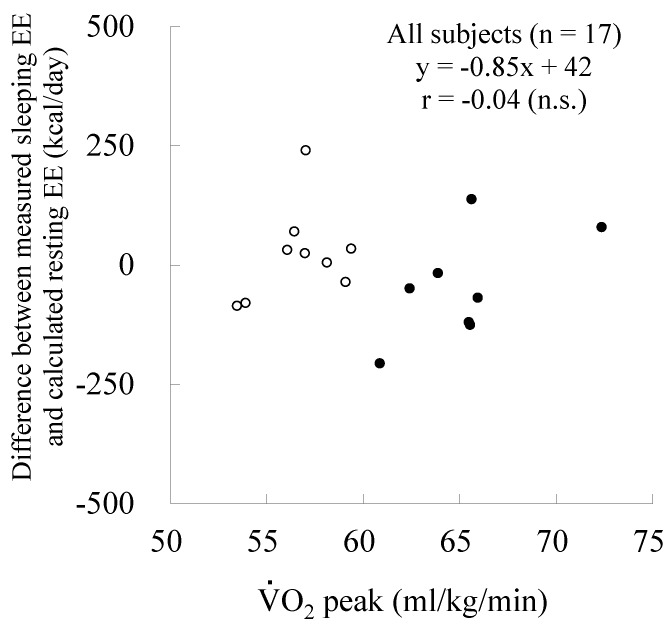
Relationship between $\overset{\cdot }{V}$O_2_peak and the difference in measured sleeping energy expenditure and calculated resting energy expenditure. EE: energy expenditure; ●: very fit runners; ○: fit runners.

The measured sleeping EE, calculated REE, and their difference in very fit runners was not significantly different from those in fit runners (Table 3, Figure 1). A significant relationship between measured sleeping EE and calculated REE was observed in the 2 groups (very fit runners: *r* = 0.79, *p* < 0.05; fit runners: *r* = 0.72, *p* < 0.05) (Figure 2). The slopes and intercepts of the two regression lines did not differ between the groups. A Bland-Altman plot showed no significant trend in both very fit and fit runners (Figure 3). Moreover, a significant correlation between $\overset{\cdot }{V}$O_2_peak and the difference in measured sleeping EE and calculated REE was not observed in all subjects (Figure 4). In addition, except for free T_4_ levels, none of the blood analysis parameters were significantly different in the very fit and fit runners (Table 4).

## 4. Discussion

In the present study, we assessed whether there is a chronic elevation in the organ-tissue sleeping metabolic rate according to the aerobic fitness level by comparing measured sleeping EE and calculated REE between very fit and fit runners and by analysing the relationship of $\overset{\cdot }{V}$O_2_peak with the difference in measured sleeping EE and calculated resting EE for all subjects. We did not observe any significant difference in the measured sleeping EE, calculated REE, and their difference, as well as in the slopes and intercepts of the two regression lines between the very fit and fit runners. Moreover, Bland-Altman analysis did not show any significant trend for very fit and fit runners. Furthermore, a significant correlation between $\overset{\cdot }{V}$O_2_peak and the difference in measured sleeping EE and calculated REE was not observed in all the subjects. These results suggest that there is no chronic elevation in the organ-tissue resting metabolic rate according to the aerobic fitness level. Moreover, the results of the present study indicated that physiological factors, such as serum TSH, total and free T_3_ and T_4_, and plasma epinephrine and norepinephrine concentrations, are not involved in the chronic elevation of the organ-tissue metabolic rate in aerobically trained populations.

A previous study with a cross-sectional design did not indicate any evidence regarding the presence of a positive relationship between REE and $\overset{\cdot }{V}$O_2_max (32–65 mL/min/kg) in young adult women, after statistically controlling for the influence of FFM [11]. In contrast, 12-week endurance exercise training (jogging and/or running for 3–4 days/week, 25–40 min/session, at 60%–80% $\overset{\cdot }{V}$O_2_max) in male participants aged 19–32 years appeared to prevent a seasonal decline in the absolute value of REE and the REE/FFM ratio; in that study, no significant change was observed in the exercise group, although significant decreases in the REE and the REE/FFM ratio were observed in the control group [14]. Furthermore, a six-month endurance training program (walking, jogging, cycling, and rowing for 3–4 days/week, 45–60 min/session, at 70%–85% maximal heart rate) in postmenopausal African-American and Caucasian women [12] and 12-month aerobic endurance training (walking or jogging for 3 days/week, 45 min/session, at 77% maximal heart rate) in previously untrained men and women [13] did not appear to affect REE and the REE adjusted for FFM. Based on the results of the present and previous studies, it appears that aerobic fitness training does not lead to an obvious chronic elevation of the organ-tissue metabolic rate.

The FFM is known as a major determinant of sleeping EE and/or REE. REE is often adjusted per unit FFM (REE/FFM) in order to compare individuals with different body sizes, and the REE/FFM ratio is used as a substitute for the organ-tissue resting metabolic rate. Moreover, the FFM is known to account for between 50% and 70% of the individual variation [10], and because ratio normalization is highly prone to confounding owing to a non-zero y-intercept for the relationship between REE and FFM, studies using appropriate regression methods such as analysis of covariance are needed to better clarify the relationship between REE and FFM. Nevertheless, REE/FFM can be used as a simple index for comparing between subjects with similar FFM. Under these conditions, it was reported that the REE/FFM in normal untrained populations is apparently smaller for individuals with a greater FFM [23]. To explain this association between FFM and REE, it was proposed that a reduction in the proportion of internal organ-tissue mass to FFM is coupled with an increase in the proportion of skeletal muscle (SM) mass in untrained individuals [4,23]. Hence, the proportion of low metabolically active tissue (e.g., SM) and high metabolically active tissue (e.g., liver and kidney), relative to the FFM, could account for the REE/FFM ratio in normal untrained populations [23]. In the present study, we observed that the sleeping EE to FFM ratio in very fit runners was not significantly different from that in fit runners. Moreover, there were no differences in the absolute values of the SM, liver, and kidney masses (which affect the REE/FFM ratio), as well as their ratios to FFM, between very fit runners and fit runners in the present study. Thus, our findings indicate that endurance exercise training does not induce a huge increase in low and high metabolically active tissue (e.g., SM, liver, and kidney) or an elevation in the REE/FFM ratio.

There are three possible limitations of this study. First, we assumed that the previously reported value of resting metabolic rate for each organ-tissue was identical among individuals. As the variation in the resting metabolic rate is correlated with many physiological and genetic factors [10], we cannot exclude the possibility that the organ-tissue energy expenditure constants have some marginal error. Hence, further research is needed to directly measure the resting metabolic rate for each organ-tissue. Second, although MRI with 1.0-cm slice thicknesses (0-cm interslice gap) is a precise and reliable method for measuring the total body SM mass in adults [19], contiguous transverse images with <0.5-cm slice thicknesses are needed to accurately estimate the masses of the brain, liver and kidneys. Moreover, as the estimation of heart mass from the body mass may contain errors, the heart mass should be more precisely assessed using ECG-triggered MRI (with an assumed metabolic rate of 440 kcal/kg/day). Third, the definition of sleeping EE and the REE calculation may be confusing. As reported in a previous study, if the sleeping EE was calculated using the formula REE × 0.935 [1] (which is the average value obtained using IHC in 71 Japanese male subjects with an average age of 36 years), we need to compare the measured- and calculated-sleeping EE in the same field. In that analysis, we noted that: (1) there was no difference between the measured- and calculated-sleeping EE in both very fit (1412 ± 179 *vs.* 1458 ± 165 kcal/day, *p* = 0.29) and fit runners (1392 ± 139 *vs.* 1369 ± 89 kcal/day, *p* = 0.49); (2) a significant relationship was observed between the measured- and calculated-sleeping EE in the two groups (very fit runners: *r* = 0.79, *p* < 0.05; fit runners: *r* = 0.72, *p* < 0.05); and (3) a Bland-Altman plot showed no significant trend in both very fit runners (*r* = 0.14, *p* = 0.75) and fit runners (*r* = 0.55, *p* = 0.12), as well as in all the subjects (*r* = 0.20, *p* = 0.43). These results also suggest that the resting metabolic rate of individual organ-tissue in aerobically trained populations with a $\overset{\cdot }{V}$O_2_max of approximately 60 mL/min/kg was similar to that in untrained subjects.

## 5. Conclusions

In conclusion, in the present study, we sought to determine whether the resting metabolic rate of individual organ-tissue in very fit adults is higher than that in fit subjects by using MRI, IHC, and blood analysis. Our findings suggest that aerobic endurance training does not have any role in the chronic elevation of the organ-tissue metabolic rate in cases with a $\overset{\cdot }{V}$O_2_max of approximately 60 mL/min/kg.

## Figures and Tables

**Table 1:** Subject characteristics.

	Very Fit Runners	Fit Runners	*p*
	*n* = 8	*n* = 9	
Age (years)	21± 1	20 ± 1	n.s.
Standing height (cm)	170.8 ± 4.7	170.9 ± 5.3	n.s.
Body mass (kg)	58.0 ± 6.7	55.0 ± 4.1	n.s.
BMI (kg/m^2^)	19.8 ± 1.7	18.8 ± 1.1	n.s.
Fat (%)	8.5 ± 0.9	10.9 ± 2.7	<0.05
Fat mass (kg)	4.9 ± 0.9	6.0 ± 1.5	n.s.
Fat-free mass (FFM) (kg)	53.0 ± 5.9	49.0 ± 4.2	n.s.
Peak heart rate (beats/min)	180 ± 10	181 ± 8	n.s.
Peak RER	1.17 ± 0.04	1.25 ± 0.08	<0.05
$\overset{\cdot }{V}$O_2_peak (mL/min)	3860 ± 320	3205 ± 292	<0.01
$\overset{\cdot }{V}$O_2_peak (mL/kg/min)	65.2 ± 3.4	56.7 ± 2.1	<0.01

BMI: body mass index; RER: respiratory exchange ratio.

**Table 2:** Body composition at the organ-tissue level.

Organ-Tissue Mass (kg)	Very Fit Runners	Fit Runners	*p*
	*n* = 8	*n* = 9	
Skeletal muscle	24.4 ± 3.2	22.5 ± 2.3	n.s.
Adipose tissue ^1^	5.9 ± 1.1	7.2 ± 1.8	n.s.
Liver	1.48 ± 0.32	1.26 ± 0.12	n.s.
Brain	1.54 ± 0.13	1.52 ± 0.10	n.s.
Heart ^2^	0.29 ± 0.02	0.28 ± 0.02	n.s.
Kidney	0.30 ± 0.08	0.30 ± 0.04	n.s.
Residual ^3^	24.0 ± 2.5	21.9 ± 2.0	n.s.

^1^ We have assumed that 85% of adipose tissue is fat and 15% of adipose tissue is the remaining calculated fat-free component, as reported by Heymsfield *et al.* [23]; ^2^ Heart mass is calculated as (22.81 × Height [m] × Body mass^0.5^ [kg] − 4.15)/1000, as reported by Ogiu *et al*. [22]; ^3^ Residual mass was calculated as body mass minus the sum of the other measured mass components.

**Table 3:** Measured sleeping energy expenditure and calculated resting energy expenditure.

	Very Fit Runners	Fit Runners	*p*
	*n* = 8	*n* = 9	
Measured sleeping EE (kcal/day)	1412 ± 179	1392 ± 139	n.s.
Calculated REE (kcal/day)	1559 ± 176	1464 ± 95	n.s.
Difference (Measured − Calculated)	−147 ± 115	−72 ± 97	n.s.

EE: energy expenditure; REE: resting energy expenditure.

**Table 4:** Blood analyses.

	Very Fit Runners	Fit Runners	*p*
	*n* = 8	*n* = 9	
TSH (μIU/mL)	1.89 ± 0.94	2.02 ± 1.21	n.s.
Total T_3_ (ng/mL)	0.96 ± 0.94	1.05 ± 0.16	n.s.
Free T_3_ (pg/mL)	2.93 ± 0.70	3.15 ± 0.33	n.s.
Total T_4_ (μg/dL)	6.54 ± 1.10	7.36 ± 0.68	n.s.
Free T_4_ (ng/dL)	1.13 ± 0.19	1.33 ± 0.79	<0.05
Epinephrine (pg/mL)	35 ± 13	59 ± 57	n.s.
Norepinephrine (pg/mL)	225 ± 112	377 ± 176	n.s.

TSH: thyroid-stimulating hormone; T3: triiodothyronine; T4: thyroxine.

## Abbreviations

EE: energy expenditure
IHC: indirect human calorimeter
REE: resting energy expenditure
FFM: fat-free mas
SM: skeletal muscle
MRI: magnetic resonance imaging
$\overset{\cdot }{V}$O_2_: oxygen uptake
$\overset{\cdot }{V}$CO_2_: carbon dioxide output
RER: respiratory exchange ratio
DXA: dual energy X-ray absorptiometry
CV: coefficient of validation
TSH: thyroid-stimulating hormone
T_3_: triiodothyronine
T_4_: thyroxine
ECG: electrocardiogram
